# Optical Genome Mapping as a New Tool to Overcome Conventional Cytogenetics Limitations in Patients with Bone Marrow Failure

**DOI:** 10.3390/genes15050559

**Published:** 2024-04-27

**Authors:** June Iriondo, Ana Gómez, Josune Zubicaray, Jorge Garcia-Martinez, Lorea Abad, Carmen Matesanz, Reyes Giménez, Almudena Galán, Alejandro Sanz, Elena Sebastián, Jesús González de Pablo, Ana de la Cruz, Manuel Ramírez, Julián Sevilla

**Affiliations:** 1 Hematology and Hemotherapy Unit, Hospital Infantil Universitario Niño Jesús, 28009 Madrid, Spain; josune.zubicaray@salud.madrid.org (J.Z.); alejandro.sanz@salud.madrid.org (A.S.); elena.sebastian@salud.madrid.org (E.S.); 2 Biomedical Research Foundation, Hospital Infantil Universitario Niño Jesús, 28009 Madrid, Spain; almudena.galan@salud.madrid.org (A.G.); jesusgpablo@gmail.com (J.G.d.P.); acruzb@salud.madrid.org (A.d.l.C.); 3 Laboratory and Clinical Analysis Department, Hospital Infantil Universitario Niño Jesús, 28009 Madrid, Spain; agomezgarcia@salud.madrid.org (A.G.); lorea.abad@salud.madrid.org (L.A.); cmbpradena@hotmail.com (C.M.); reyesgimenez18@gmail.com (R.G.); manuel.ramirez@salud.madrid.org (M.R.); 4 Pediatric Onco-Hematology Department, Hospital Infantil Universitario Niño Jesús, 28009 Madrid, Spain; xurde.garcia.martinez@gmail.com; 5 Health Research Institute at Hospital de La Princesa (IIS-Princesa), 28006 Madrid, Spain

**Keywords:** bone marrow failure, cytogenetics, optical genome mapping, aplastic anemia, Fanconi anemia

## Abstract

Cytogenetic studies are essential in the diagnosis and follow up of patients with bone marrow failure syndromes (BMFSs), but obtaining good quality results is often challenging due to hypocellularity. Optical Genome Mapping (OGM), a novel technology capable of detecting most types chromosomal structural variants (SVs) at high resolution, is being increasingly used in many settings, including hematologic malignancies. Herein, we compared conventional cytogenetic techniques to OGM in 20 patients with diverse BMFSs. Twenty metaphases for the karyotype were only obtained in three subjects (15%), and no SVs were found in any of the samples. One patient with culture failure showed a gain in chromosome 1q by fluorescence in situ hybridization, which was confirmed by OGM. In contrast, OGM provided good quality results in all subjects, and SVs were detected in 14 of them (70%), mostly corresponding to cryptic submicroscopic alterations not observed by standard techniques. Therefore, OGM emerges as a powerful tool that provides complete and evaluable results in hypocellular BMFSs, reducing multiple tests into a single assay and overcoming some of the main limitations of conventional techniques. Furthermore, in addition to confirming the abnormalities detected by conventional techniques, OGM found new alterations beyond their detection limits.

## 1. Introduction

Cytogenetic studies are essential in the diagnostic workup and follow-up of patients with acquired or congenital bone marrow failure syndromes (BMFSs). On the one hand, at diagnosis, karyotype abnormalities may have prognostic significance or even provide diagnostic clues for suspicion of germline variants (such as monosomy 7 in SAMD9/SAMD9L syndromes). On the other hand, they are important in the follow-up for monitorization of clonal evolution and transformation [1].

Conventional cytogenetic techniques include mainly karyotype analysis, fluorescence in situ hybridization (FISH), and chromosomal microarrays (CMAs), and the cytogenetic analysis of the samples usually relies on a combination of them. However, these techniques have many limitations. CMAs are not able to detect balanced translocations, FISH is necessarily targeted and needs previous knowledge of the genes to be studied, and though karyotype analysis is able to examine chromosomal structural variants (SVs) throughout the entire genome, its resolution is low (>5 mega base pairs -mbp-) and is technically complex [2]. Moreover, in hypocellular bone marrow samples, it is often very challenging to obtain enough metaphases for karyotyping after cell culture, which significantly impacts on the quality and reliability of this study.

In recent years, Optical Genome Mapping (OGM) has emerged as a novel cytogenomic technology capable of detecting all types of chromosomal structural rearrangements (insertions, duplications, deletions, inversions, and translocations) at much higher resolution than standard techniques [2]. According to information provided by the manufacturer, at 300× effective coverage, OGM is able to detect translocations and inversions > 70 kilo base pairs (kbp), deletions > 25 kbp, insertions 5–50 kbp, and duplications 100–300 kbp at 5% allele fraction with a 90% sensitivity.

Briefly, its technical fundament is based on labeling ultra-high-molecular-weight DNA with fluorescence-marked short nucleotide sequence motifs, which results in an identifiable sequence-specific pattern of labels across the human genome that is then longitudinally aligned and imaged with a high-resolution camera. Finally, algorithms convert the images into molecules that are assembled into consensus genome maps for their posterior analysis and comparison to a reference genome or between samples [2]. 

OGM is being increasingly implemented in cytogenetics laboratories and its use has been explored in diverse settings, including prenatal or postnatal constitutional studies, solid tumors, and diverse hematologic malignancies [3,4,5,6,7,8,9,10]. However, to our knowledge, there are no reports about its applicability in BMFSs to date.

In this work, we aimed to describe the performance of OGM in comparison to conventional cytogenetics in bone marrow (BM) samples of 20 patients with BMFSs in our center.

## 2. Materials and Methods

Fresh BM aspirate samples from 20 pediatric patients with suspected or known BMFSs collected between April 2021 and November 2022 at the Hospital Infantil Universitario Niño Jesús (HIUNJ) in Madrid (Spain) were included in the study. 

BM cytogenetic studies were ordered by treating physicians as part of routine diagnostic or follow-up explorations. OGM was performed in those in which there was excess material after conventional techniques were performed.

Written informed consent for the BM aspiration and/or biopsy and for sedation for the procedure was obtained from each patient or their legal representative. This study was approved by the HIUNJ ethics committee (internal code R-0034/23).

### 2.1. Cytogenetic Studies

Regarding conventional cytogenetics, karyotype analysis was performed by G banding, and following national and international recommendations, a minimum of 20 metaphases were analyzed whenever possible [11]. FISH was performed on interphase nuclei, and, in all cases, 200 interphase nuclei were analyzed by direct fluorescence microscopy [12]. The specific procedures and probes used in each case are detailed in the Methods section of the Supplementary Materials. 

For Optical Genome Mapping (OGM), samples were analyzed using the Bionano Genomics Saphyr platform (Bionano Genomics Inc., San Diego, CA, USA) following the manufacturers’ protocols. The quality control targets for the analysis were a >300× effective coverage of the genome, >70% mapping rate, 14 to 17 label density (labels per 100 kbp), and >230 kbp N50 (of molecules > 150 kbp). The data analysis was performed using the “Rare Variant Analysis” (RVA) algorithm and results were visualized with the Bionano Access software (v1.6 Bionano Genomics). In addition, a coverage-based algorithm enabled the detection of large CNVs and aneuploidies. 

The confidence scores (range 0–1) for SVs applied for the RVA analysis were the following: 0 for insertions, 0 for deletions, 0.7 for inversions, −1 for duplication and 0.3 for intra-fusion and 0.65 for inter-translocation. After applying the described filters, SVs larger than 100 kbp were reported (SVs < 100 kbp were reported if they affected clinically relevant genes). Each alteration was compared to Bionano’s human control sample SV database, which contains variants collected from ethnically diverse mapped human genomes with no reported disease phenotypes. The detected variants were further curated and then manually inspected to identify true calls, as detailed in the Methods section of the Supplementary Materials. All samples were analyzed and reported by a single experienced cytogeneticist of our center.

### 2.2. Data Analysis

Data from quantitative variables are presented as the median and the range or interquartile range (IQR), and qualitative variables are expressed in frequencies and percentages (%). 

Regarding the SV calls by OGM, they were posteriorly crosschecked with the Genome Aggregation Database (gnomAD) SV v4 and processed with R statistical computing system v4.3.2 for a population frequency estimation, with the aim of providing additional information to the VAF to deduct a potential germline or somatic origin of the variants. Since several regions were found in the gnomAD database for each of the detected SVs, the maximum, minimum, mean, and a weighted mean were calculated for each of them according to the size of the fragment that colocalizes with the SV.

## 3. Results

A total of 20 samples of 20 pediatric patients were included in the analysis. Median age was 9 years (IQR 5–13.75), and the most frequent diagnosis was Fanconi anemia (FA) in 13 patients (65%), followed by aplastic anemia (AA) and neutropenia in 5 (25%) and 2 (10%) patients, respectively. One of the patients with neutropenia was known to have a variant in the JAGN1 gene by sequencing techniques, associated with severe congenital neutropenia. In the others, no germline cause was identified. Acquired aplastic anemia was diagnosed in patients with no characteristic phenotypic findings and a negative next-generation sequencing study for inherited BMFSs. Patients’ baseline characteristics are shown in Table 1.

Regarding conventional cytogenetics, the target of 20 metaphases for G banding was only achieved in three of the 20 patients (15%), with a median of 10 metaphases analyzed (IQR 8.5–15), and there was one case in which no metaphases were obtained at all. No SVs were found in any of the samples. In the case of FISH, there was only one positive case (5%) in an FA patient, in which a gain in chromosome 1(q21–22) was found in 7% of the studied nuclei and was, in fact, the sample in which no metaphases were obtained. No other chromosomal rearrangements were found in the rest of the cases (Table 2). 

Strikingly, SVs were identified by OGM in 14 of the 20 samples (70%) applying the filtering criteria mentioned above. SVs were found in 12 of the 13 FA patients (92.3%), 2 of the 5 AA patients (40%), and in none of the patients with neutropenia (Table 2). A total of 21 SVs were found, with a median of 1 SV per patient (range 0–3). The assay showed eight deletions, five insertions, and eight duplications, and all of them were small SVs < 500 kbp with variant allele frequencies (VAF) ranging from 7% to 59%, which would be beyond the purview of karyotype analysis. All samples fulfilled the pre-stablished quality control criteria on the first try, with a median average label density of 15.49 labels/100 kbp (range 14–17), median mapping rate 89.35% (range 78.6–93%) and a median effective coverage of 433× (range 300–464×). There were no incidences in the processing or during the analysis. Specific details such as size, VAF, genomic coordinates and the quality matrix of each sample are shown in Supplementary Table S1.

**Figure 1:**
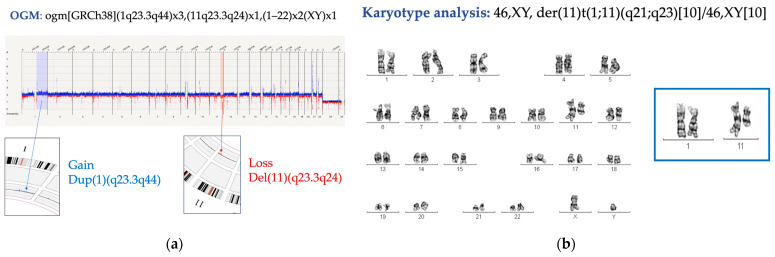
Cytogenetic results of subject number 8. (**a**) Representation of Copy Number Variant (CNV) analysis results by optical genome mapping (OGM), in which a gain in chromosome 1q (blue arrow) and a loss in chromosome 11q (red arrow) are observed. (**b**) Karyotype in a subsequent bone marrow aspirate sample, in which a translocation between chromosome 1 and chromosome 11 is observed with a gain in chromosome 1q (highlighted in the blue box).

Interestingly, in the case in which no metaphases were obtained after culture and a 1q gain was observed by FISH, OGM found the 1q gain and also detected a loss in chromosome 11 (q23) with a clonal level of 7%. In a subsequent BM analysis in which metaphases were obtained, the above-mentioned SVs were found to be related in the form of a translocation between said chromosomes (Figure 1a,b). OGM was not able to correlate both alterations due to the fact that the breakpoint of the 1q gain is located in the peri-centromeric region, a difficult-to-map area.

**Figure 2:**
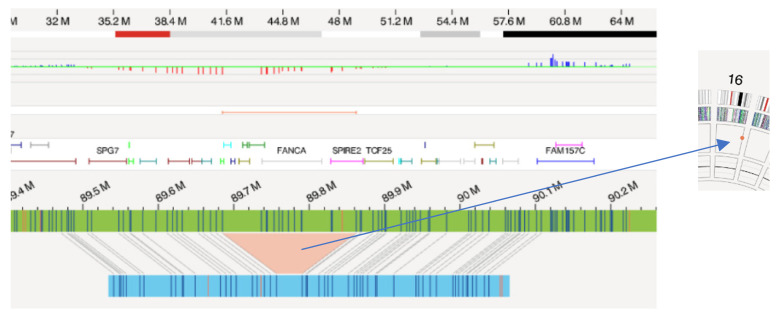
Deletion in chromosome 16q (FANCA gene) from subject number 2 as observed by Optical Genome Mapping (OGM). The orange area represents the segment of the reference genome (in green) that has been lost in the patient’s chromosome (in blue), represented as a red dot in the circus plot (blue arrow).

Furthermore, in another FA patient (ID 2), OGM was able to detect a small deletion of 143 kbp in chromosome 16q with a VAF of 56%, which corresponds to the deletion of exons 1–43 of the FANCA gene (Figure 2), which, together with the c.1115_1118del, p.(Val372Alafs*42) variant found in exon 13 of the FANCA gene by sequencing techniques, confers the FA diagnosis upon the patient.

All regions affected by the different SVs were explored for potential genes of interest in patients with BMFSs, with the above-mentioned FANCA being the only one. No other genes were regarded as clinically relevant in BMFSs in the rest of the samples.

Although outside the aim of this study, we analyzed the results obtained by OGM to evaluate if the technique could be used to differentiate the somatic or germline origin of the observed SVs. If so, it would be of great help, avoiding the need of studies in non-hematopoietic tissue. However, the VAFs provided by OGM together with the crosschecking of the SVs against the gnomAD database could not help for an initial approximation. We observed that 6 of the 21 (28.57%) SVs presented at clonal levels < 40%, with an estimated frequency in the general population < 1% in 5 of them (no matching variants were found in gnomAD for the other SV), which would support a somatic origin. Interestingly, there was only one SV (4.76%) with an estimated frequency > 1% in the general population, corresponding to a duplication with a 55% VAF in subject 9, suggesting a germline origin. Nonetheless, as mentioned, specific studies should be carried out to address this issue. Detailed results are presented in Table S1 of the Supplementary Materials.

## 4. Discussion

As mentioned before, cytogenetic studies are of great importance in patients with BMFSs. For instance, patients with FA present with chromosomal instability and high levels of chromosome breaks induced by DNA interstrand crosslinkers, which predisposes them to BMF and myelodysplastic neoplasm/acute myeloid leukemia (MDS/AML). In a recent study published by Sebert et al. in which they analyzed 335 FA patients from the French registry, the main somatic lesions involved SVs rather than point mutations, the most frequent alterations being partial or total 1q gains (51.6%), partial 3q gains (40.3%), partial loss of 7q or monosomy 7 (30.6%), and lesions at 21q22 altering the *RUNX1* locus (22.6%). They found that 1q trisomy is the most frequent and early event in the natural history of bone marrow evolution in FA, but it is not transforming per se, and additional oncogenic events like 3q gains or del7q/-7 are needed to evolve to MDS/AML [14]. On the other hand, in the case of acquired conditions, cytogenetic abnormalities play a major role in the differential diagnosis between aplastic anemia and MDS in pediatric patients, in which sole morphological distinction is often challenging due to the fact that the majority of childhood MDS are hypocellular [15,16,17]. Monosomy 7 is the most common cytogenetic abnormality in childhood MDS (seen in approximately 20–30% of the cases) and indicates risk of progression to AML, followed by trisomy 8 and trisomy 21 [16,18,19]. In a study in which 118 hypocellular bone marrow biopsies from adults (76) and children (42) with an initial diagnosis for AA were evaluated, 10 of the 42 children (24%) presented with a compatible histology of MDS, and 6 of them had conclusive cytogenetic studies at diagnosis. Out of the 42 children, 6 progressed to MDS/AML, with only 1 case of progression within the MDS group, concluding that histology is not predictive of progression [17]. Thus, these studies demonstrate the need for a comprehensive evaluation of the cytogenetics in patients with diverse forms of bone marrow failure.

Unfortunately, in our experience, karyotype failures or not obtaining the recommended 20 metaphases are frequent in patients with a hypocellular bone marrow, which limits the reliability of the technique. However, published information regarding this issue is scarce. In the above-mentioned study by Marchesi et al., of the 118 samples, the authors refer that cytogenetics were conclusive in 84 (combining karyotype and FISH); however, they do not specify the exact number of karyotype failures nor the amount of metaphases analyzed in each sample, but in the reported karyotype results of the 12 patients that progressed, 6 were non-evaluable due to hypocellularity, and less than 20 metaphases are present in the rest [17]. Interestingly, in Sebert and colleagues’ study, CGH and SNP arrays were used as alternative techniques to karyotyping by G-banding, which is a much more extended and available technique in the clinical setting. We believe that these relevant reports should serve to highlight that failure to reach conclusive results with G banding in clinical laboratories is a general issue that needs to be addressed.

OGM was implemented in the clinical laboratory of our institution in 2021 with the aim of substituting standard cytogenetic techniques in every setting, such as constitutional studies, different malignancies, and other conditions, including bone marrow failure syndromes. Surprisingly, we observed that we were able to reach the required quality control targets in most cases, including hypocellular marrows. Its clinical utility was then explored in 97 samples of patients with acute lymphoblastic leukemia (the most prevalent condition in which cytogenetic studies are performed in our center), demonstrating full concordance with conventional techniques. Moreover, besides confirming the alterations found by standard assays, OGM detected additional variants in many cases [20]. Hence, it is now included as standard practice for this disease.

As mentioned before, OGM’s utility has also been explored by many other groups in different conditions and has demonstrated excellent results and concordance with traditional cytogenetic analysis [2,4,5,7,9,21,22,23]. For example, in a clinical validation study in which 92 sample runs were included (belonging to 69 samples, 59 hematological neoplasms and 10 healthy controls), OGM showed a sensitivity of 98.7%, specificity of 100% and an accuracy of 99.2% in comparison to standard techniques (karyotype analysis, FISH, and chromosomal microarray), with a 100% first-pass rate regarding the quality control metrics [2]. Furthermore, Yang et al. explored the clinical value of structural variant profiling by OGM in 101 adult patients with newly diagnosed MDS. Of those, 62% presented at least one clonal cytogenetic alteration by karyotype analysis (median 1, range 0–12), and OGM found clinically significant SVs (defined as SVs that overlap the coding region of a gene/chromosome locus implicated in myeloid neoplasms) in 70% (median 1, range 0–47). Out of 4030 unique somatic variant calls, 383 were considered clinically significant, and, of those, 224 were only apparent by OGM, either because of sub-microscopic or cryptic nature beyond the detection limit of G banding or poor chromosomal morphology in complex karyotypes. Furthermore, OGM generated successful sequence patterns in the two patients in which no metaphases were obtained and its higher resolution allowed clarification of marker chromosomes or additional material of unknown origin. OGM findings caused a change in R-IPSS risk score in 17% of the patients [9].

In the present study, given the lack of publications addressing OGM’s applicability in BMFSs, we decided to explore its performance in this rare group of patients. We included patients in which both conventional cytogenetics (karyotype analysis and FISH) and OGM were performed when OGM became available in our center. Interestingly, we detected SVs in the majority of the subjects, most beyond the detection limit of the karyotype or out of the FISH target genes or breakpoints.

None of the patients have developed overt myelodysplastic syndrome or acute myeloid leukemia so far, and most of the detected SVs probably belong to small constitutional alterations with no clinical significance given their characteristics and clonal values. However, to clarify, that is beyond the scope of this work, but we believe it will pave the way for future studies. In accordance with Behrens et al., we believe they should be considered as “aberrations of indeterminate potential” until further studies clarify their role [24].

As demonstrated in this work despite the small number of samples, OGM offers multiple advantages over conventional cytogenetics; on the one hand, owing to its ability to detect most types of SVs, it can potentially reduce multiple tests into a single assay, considerably reducing resources and turn-around time. Furthermore, it detects SVs at much higher resolution than standard techniques and in a genome-wide setting, which provides the possibility of discovering new undescribed aberrations and to identify different partner genes cryptic to standard techniques. Most importantly, in patients with hypocellular BMFs, in whom obtaining enough metaphases for karyotype analysis results challenging, OGM was capable of obtaining good quality results in all cases, which overcomes one of the main difficulties of the classic technique and highlights its applicability in this population.

However, OGM has some limitations. One of the most relevant limitations is the difficulty to assess SVs with breakpoints in the “poorly covered areas”, such as acrocentric centromeres, short arms of acrocentric chromosomes, telomeres and heterochromatin regions [22]. In fact, in FA, the proximal breakpoints at chromosome 1q are most frequently located at the centromeric/pericentromeric repeated region; in contrast, in the other unbalanced translocations such as 3q+ they spread along the chromosome arms [14]. Other authors have also reported failure of OGM at identifying low subclones (<5% VAF), mainly those involving whole chromosomes, probably due to the proliferative advantage of certain clones during culture, which makes them apparent for conventional techniques [9,22,25]. Nonetheless, that threshold can probably be reduced by increasing the coverage [25]. Furthermore, karyotype and FISH studies also have interest as complement to OGM or other global techniques in the identification and follow-up of low subclones given their single-cell based analysis.

## 5. Conclusions

In conclusion, we believe OGM ensures complete, reliable and good quality results in patients with BMFSs and may reduce multiple tests into a single assay, solving the main problems of conventional techniques. Furthermore, it offers shorter turn-around times, which is of great importance in the clinical setting. However, standard cytogenetics will probably still have a role in the future for cases in which OGM cannot accurately describe certain SVs in poorly covered areas or in cases with low subclones. Finally, we emphasize the need for future larger studies that will consolidate our findings, will explore the applicability of OGM in other bone marrow failure syndromes, and will help to define the clinical significance of the “aberrations of indeterminate potential”.

## Figures and Tables

**Table 1:** Patients’ baseline characteristics.

	Number of Patients N = 20	%
Age (years) - Median (IQR)	9 (5–13.7)	
Sex: - Female - Male	10 10	50 50
Diagnosis: - Fanconi Anemia - Aplastic Anemia - Neutropenia	13 5 2	65 25 10
BM ^1^ hypocellularity [13]: - Severe (<25%) - Moderate (25–50%) - Mild/normal (>50%) - Unknown	7 8 3 2	35 40 15 10

^1^ BM: bone marrow.

**Table 2:** Results of cytogenetic studies. FISH: Fluorescent In situ Hybridization. OGM: Optical Genome Mapping.

Sample ID	Diagnosis	Karyotype	FISH	OGM Results According to ISCN Ogm[GRCh38]
ID 1	Fanconi Anemia	46,XX[13]	negative	(1–22,X)x2
ID 2	Fanconi Anemia	46,XX[10]	negative	16q24.3(89685110_89862359)x1∼2
ID 3	Fanconi Anemia	46,XX[10]	negative	14q32.33(105710125_105754681)x2∼3 21p11.2(10326071_10766790)x2∼3
ID 4	Fanconi Anemia	46,XY[8]	negative	7q11.23(74,869,402_75214597)x1∼2 9q34.3(135271879_135407027)x2∼3 16p12.3(18262064_18751754)x1
ID 5	Fanconi Anemia	46,XY[12]	negative	13q21.33(71965822_72074078)x1
ID 6	Fanconi Anemia	46,XX[10]	negative	12q24.32(127160989_127467513)x2∼3
ID 7	Fanconi Anemia	46,XX[12]	negative	2p12(81983068_82084517)x2∼3
ID 8	Fanconi Anemia	0 metaphases	nuc ish(CDKN2C x2, CKS1B x3) [14/200]	1q23.3q44(163150928_248943333)x2∼3 11q23.3q24(115535614_121492796)x1 7p12.1(53380529_53527833)x1
ID 9	Fanconi Anemia	46,XY[15]	negative	7q11.23(76804855_77042022)x2∼3 16q23.1(75525291_75543486)x2∼3
ID 10	Fanconi Anemia	46,XY[10]	negative	10q11.21q11.23(47461135_47776804)x2∼3
ID 11	Fanconi Anemia	46,XY[8]	negative	4q13.2(68625142_68669119)x2∼3
ID 12	Fanconi Anemia	46,XY[15]	negative	1q43(238591073_238614424)x2∼3 7q11.21(65486487_65566699)x2∼3
ID 13	Fanconi Anemia	46,XY[20]	negative	1p34.1(45493706_45606883)x2∼3
ID 14	Acquired Aplastic Anemia	46,XX[5]	negative	1q31.3(195372266_195473816)x1
ID 15	Acquired Aplastic Anemia	46,XX[10]	negative	2p24.2(16866735_17121008)x1
ID 16	Acquired Aplastic Anemia	46,XX[20]	negative	(1–22,X)x2
ID 17	Acquired Aplastic Anemia	46,XX[20]	negative	(1–22,X)x2
ID 18	Acquired Aplastic Anemia	46,XX[15]	negative	(1–22,X)x2
ID 19	Severe Congenital Neutropenia	46,XY[8]	negative	(1–22,X)x2

## Data Availability

All data relevant to the study are included in the article or as Supplementary Materials.

## Supplementary Materials

The following supporting information can be downloaded at: https://www.mdpi.com/article/10.3390/genes15050559/s1, Supplementary Methods section; Table S1: Specific details of each structural variant detected by Optical Genome Mapping.
