# Supplementary material for: Optical Genome Mapping as a New Tool to Overcome Conventional Cytogenetics Limitations in Patients with Bone Marrow Failure
Source: Genes (Basel). 2024 Apr 27;15(5):559. doi: 10.3390/genes15050559 (PMC11121707; doi:10.3390/genes15050559)
Supplement: Supplementary file 1 [file genes-15-00559-s001.zip › genes-2973724-supplementary.pdf]

SUPPLEMENTARY METHODS

BM cellularity was evaluated by local pathologists in those patients with available biopsy specimens, and classified as severe if <25%, moderate 25-50% or mild/normal >50%.(1)

Cytogenetic studies:

Karyotype analysis by G banding:

Following local protocols, 0,5 mililiters (mL) of the first BM aspirate tube were used for this technique. In all cases, direct cell cultures in Roswell Park Memorial Institute (RPMI) 1640 medium were performed on the same day of sample obtention. 250µL of colchicine (Colcemid®) were added to depolymerize the microtubules and cells were then subjected to a hypotonic shock with potassium chloride (KCl 0,075M). Finally, cells were pre-fixed with Carnoy's solution, extended and, after ageing by heat (56ºC for 24 hours), stained with Leishman's Stain for G banding.

Following national and international recommendations, a minimum of 20 metaphases were analyzed whenever possible.(2) All samples were analyzed and reported by a single experienced cytogeneticist of our center.

Fluorescent In Situ Hybridization (FISH):

FISH was performed on interphase nuclei. Fresh BM aspirate sample extensions were prepared and dehydrated in increasing concentrations of ethanol (70º, 85º and 100º), and then fixed with a 0,005% pepsin solution. Preparations were then observed by direct microscopy (Leica®) to select the area for hybridization, and after adding the selected probes they were heated at 70ºC for 6 minutes for denaturation (Izasa Scientific). Finally, the samples were incubated for a minimum of 16 hours at 37ºC for hybridization in an incubator shaker (New Brunswick Scientific™) before analysis.

In Fanconi Anemia patients, we searched for chromosomal rearrangements in chromosomes 1, 3 and 7 by using the following probes: XL CDKN2C(1p32.3)/CKS1B(1q21-22) Dual color, LSI RPN1(3q21.3)/MECOM(3q26.2) Dual color fusion and XL 7q22/7q36/CEP 7 Tricolor. In the rest of the cases the probes LSI EGR1(5q31)/D5S721, D5S23 Dual color, XL 7q22/7q36/CEP 7 Tricolor and CEP 8 Spectrum Orange were used for chromosomes 5, 7 and 8, respectively.

In all cases, 200 interphase nuclei were analyzed by direct fluorescence microscopy (Leica®). The positivity threshold was 10% for monosomy, 5% for trisomy, deletions and gains, and 1-5% for translocations according to national guidelines and local experience.(3)

Optical Genome Mapping (OGM):

Samples were analyzed using the Bionano Genomics Saphyr platform (Bionano Genomics Inc., San Diego, CA) following the manufacturers' protocols. First, nucleated white blood cells were isolated from the BM aspirate sample by ficoll density gradient centrifugation. Posteriorly, ultra-high-molecular-weight DNA was isolated and labeled according to the manufacturers' instructions. Then, labeled DNA was loaded onto the Saphyr chips and electrophoretically linearized for optical imaging and posterior analysis.

The quality control targets for the analysis were a >300x effective coverage of the genome, >70% mapping rate, 14 to 17 label density (labels per 100 kbp), and >230 kbp N50 (of molecules >150 kbp).

The data analysis was performed using the "Rare Variant Analysis" (RVA) algorithm and results were visualized with the Bionano Access software (v1.6 Bionano Genomics). The RVA allows for SV detection by looking for mismatches between molecules and the reference genome (GRCh38/hg38), by comparing the molecules directly to the reference. In addition, a coverage-based algorithm enabled the detection of large CNVs and aneuploidies.

The confidence scores (range 0–1) for SVs applied for the RVP analysis were the following: 0 for insertions, 0 for deletions, 0.7 for inversions, -1 for duplication and 0.3 for intra-fusion and 0.65 for inter-translocation. After applying the described filters, SVs larger than 100 Kbp were reported (SVs <100 Kbp were reported if they affected clinically relevant genes). Each alteration was compared to Bionano's human control sample SV database, which contains variants collected from ethnically diverse mapped human genomes with no reported disease phenotypes. To filter the variants, we first selected those that were present in 0% of the population database. Secondly, to exclude variants located in high-variance regions, we evaluated only structural variations (SVs) in non-masked regions. Thirdly, we reported variants that were present in at least 5 molecules. Finally, each alteration was manually inspected to identify true calls. All samples were analyzed and reported by a single experienced cytogeneticist of our center.

Supplementary Table 1. Specific details of each structural variant detected by Optical Genome Mapping. SV: Structural Variant. OGM: Optical Genome Mapping.

<table>
<tr><th rowspan="2">Subject ID</th><th rowspan="2">SV type</th><th rowspan="2">Size (Kbp)</th><th rowspan="2">OGM results according to ISCN ogm[GRCh38]</th><th rowspan="2">VAF (%)</th><th rowspan="2">Average label density (>= 150 kbp)</th><th rowspan="2">Map rate (>= 150 kbp) (%)</th><th rowspan="2">Effective coverage</th><th colspan="4">Estimation of SV frequency in general population</th></tr>
<tr><th>Weighed average</th><th>Mean</th><th>Maximum</th><th>Minimum</th></tr>
<tr><td>ID 1</td><td>No SVs found</td><td>-</td><td>(1-22,X)x2</td><td>-</td><td>17</td><td>84</td><td>407</td><td>-</td><td>-</td><td>-</td><td>-</td></tr>
<tr><td>ID 2</td><td>deletion</td><td>143.650</td><td>16q24.3(89685110_89862359)x1~2</td><td>56</td><td>16.30</td><td>89.7</td><td>443</td><td>0.017 %</td><td>0.335 %</td><td>18.152 %</td><td>0.001 %</td></tr>
<tr><td>ID 3</td><td>insertion</td><td>120.785</td><td>14q32.33(105710125_105754681)x2~3</td><td>21</td><td rowspan="2">16.80</td><td rowspan="2">84</td><td rowspan="2">331</td><td colspan="4">No matching found in gnomAD for insertions</td></tr>
<tr><td>ID 3</td><td>duplication</td><td>440.719</td><td>21p11.2(10326071_10766790)x2~3</td><td>51</td><td>0.524 %</td><td>0.246 %</td><td>17.505 %</td><td>0.001 %</td></tr>
<tr><td>ID 4</td><td>deletion</td><td>255.964</td><td>7q11.23(74,869,402_75214597)x1~2</td><td>23</td><td rowspan="3">16.30</td><td rowspan="3">90</td><td rowspan="3">440</td><td>0.111 %</td><td>0.092 %</td><td>2.21 %</td><td>0.001 %</td></tr>
<tr><td>ID 4</td><td>duplication</td><td>135.148</td><td>9q34.3(135271879_135407027)x2~3</td><td>59</td><td>0.032 %</td><td>0.192 %</td><td>2.492 %</td><td>0.001 %</td></tr>
<tr><td>ID 4</td><td>deletion</td><td>489.690</td><td>16p12.3(18262064_18751754)x1</td><td>51</td><td>0.479 %</td><td>2.613 %</td><td>30.966 %</td><td>0.001 %</td></tr>
</table>

ID 5	deletion	105.402	13q21.33(71965822_72074078)x1	45	14.51	89	435	0.006 %	0.015 %	0.089 %	0.001 %
ID 6	duplication	306.524	12q24.32(127160989_127467513)x2~3	45	15	93	464	0.004 %	0.005 %	0.027 %	0.001 %
ID 7	duplication	101.449	2p12(81983068_82084517)x2~3	46	15.36	92.9	456	0.009 %	0.091 %	0.618 %	0.001 %
ID 8	duplication	24,943.777	1q23.3q44(163150928_248943333)x2~3	7	15.72	88	422	0.017 %	0.657 %	90.897 %	0.001 %
ID 8	deletion	5,957.182	11q23.3q24(115535614_121492796)x1	7				0.032 %	0.063 %	0.785 %	0.001 %
ID 8	deletion	126.777	7p12.1(53380529_53527833)x1	49				0.035 %	0.102 %	21.562 %	0.001 %
ID 9	duplication	237.167	7q11.23(76804855_77042022)x2~3	55	15.17	91	300	1.446 %	6.152 %	67.076 %	0.001 %
ID 9	insertion	122.800	16q23.1(75525291_75543486)x2~3	44				0.008 %	0.008 %	0.008 %	0.008 %
ID 10	duplication	315.669	10q11.21q11.23(47461135_47776804)x2~3	51	15.26	90	443	0.388 %	5.168 %	21.367 %	0.001 %
ID 11	insertion	102.354	4q13.2(68625142_68669119)x2~3	30	15.78	84	416	0.062 %	0.019 %	0.078 %	0.001 %
ID 12	insertion	126.666	1q43(238591073_238614424)x2~3	59	15.48	92.5	449	0.037 %	0.09 %	0.236 %	0.002 %
ID 12	insertion	211,632	7q11.21(65486487_65566699)x2~3	36				0.805 %	3.726 %	37.073 %	0.001 %

ID 13	duplication	113,177	1p34.1(45493706_45606883)x2~3	52	15.74	88.7	431	0.001 %	0.01 %	0.237 %	0.001 %
ID 14	deletion	101	1q31.3(195372266_195473816)x1	47	15.50	78.6	386	0.003 %	0.052 %	0.665 %	0.001 %
ID 15	deletion	247,495	2p24.2(16866735_17121008)x1	51	15.69	92.4	460	0.032 %	0.032 %	0.462 %	0.001 %
ID 16	No SVs found	-	(1-22,X)x2	-	14	80	402	-	-	-	-
ID 17	No SVs found	-	(1-22,X)x2	-	15.90	91.4	446	-	-	-	-
ID 18	No SVs found	-	(1-22,X)x2	-	15.20	86.1	419	-	-	-	-
ID 19	No SVs found	-	(1-22,X)x2	-	14.89	85.3	300	-	-	-	-
ID 20	No SVs found	-	(1-22)x2,(XY)x1	-	15.07	91.8	451	-	-	-	-

References:

1. Woessner, S.; Florensa L. *La citologia óptica en el diagnóstico hematológico [Optical Cytology in Hematologic diagnosis].* 5th ed. Acción Médica; **2006**.

2. Rack, K.A.; van den Berg, E.; Haferlach, C.; Beverloo, H.B.; Costa, D.; Espinet, B.; Foot, N.; Jeffries, S.; Martin, K.; O'Connor, S.; et al. European recommendations and quality assurance for cytogenomic analysis of haematological neoplasms. Leukemia 2019, 33, 1851–1867. https://doi.org/10.1038/s41375-019-0378-z.

3. Aloui N, Cuatrecasas E, Garrido MA, Jiménez M, Pujol N, Salido M, .; Jiménez, M.; Pujol, N.; Salido, M.; Verge, A. *Análisis citogenómicos aplicados a neoplasias hematológicas: recomendaciones preanalíticas, analíticas y postanalíticas [Cytogenomic analysis applied to hematologic neoplasms: pre-analytic, analytic and post-analytic recommendations]*. 1st ed. Espinet B, Blanco ML, Costa D, Cuatrecasas E, Ruiz-Xivillé N, editors. Sociedad Española de Hematología y Hemoterapia: Madrid, Spain, 2021.
